# Development of an automated artificial intelligence-based system for urogenital schistosomiasis diagnosis using digital image analysis techniques and a robotized microscope

**DOI:** 10.1371/journal.pntd.0012614

**Published:** 2024-11-05

**Authors:** Carles Rubio Maturana, Allisson Dantas de Oliveira, Francesc Zarzuela, Edurne Ruiz, Elena Sulleiro, Alejandro Mediavilla, Patricia Martínez-Vallejo, Sergi Nadal, Tomàs Pumarola, Daniel López-Codina, Alberto Abelló, Elisa Sayrol, Joan Joseph-Munné

**Affiliations:** 1 Microbiology Department, Vall d’Hebron University Hospital, Vall d’Hebron Research Institute (VHIR), Barcelona, Spain; 2 Department of Microbiology and Genetics, Universitat Autònoma de Barcelona (UAB), Barcelona, Spain; 3 Computational Biology and Complex Systems Group, Physics Department, Universitat Politècnica de Catalunya (UPC), Castelldefels, Spain; 4 CIBERINFEC, ISCIII—CIBER de Enfermedades Infecciosas, Instituto de Salud Carlos III, Madrid, Spain; 5 Database Technologies and Information Management Group, Service and Information Systems Engineering Department, Universitat Politècnica de Catalunya (UPC), Barcelona, Spain; 6 Tecnocampus, Universitat Pompeu Fabra, Mataró, Spain; George Washington University School of Medicine and Health Sciences, UNITED STATES OF AMERICA

## Abstract

**Background:**

Urogenital schistosomiasis is considered a Neglected Tropical Disease (NTD) by the World Health Organization (WHO). It is estimated to affect 150 million people worldwide, with a high relevance in resource-poor settings of the African continent. The gold-standard diagnosis is still direct observation of *Schistosoma haematobium* eggs in urine samples by optical microscopy. Novel diagnostic techniques based on digital image analysis by Artificial Intelligence (AI) tools are a suitable alternative for schistosomiasis diagnosis.

**Methodology:**

Digital images of 24 urine sediment samples were acquired in non-endemic settings. *S*. *haematobium* eggs were manually labeled in digital images by laboratory professionals and used for training YOLOv5 and YOLOv8 models, which would achieve automatic detection and localization of the eggs. Urine sediment images were also employed to perform binary classification of images to detect erythrocytes/leukocytes with the MobileNetv3Large, EfficientNetv2, and NasNetLarge models. A robotized microscope system was employed to automatically move the slide through the X-Y axis and to auto-focus the sample.

**Results:**

A total number of 1189 labels were annotated in 1017 digital images from urine sediment samples. YOLOv5x training demonstrated a 99.3% precision, 99.4% recall, 99.3% F-score, and 99.4% mAP0.5 for *S*. *haematobium* detection. NasNetLarge has an 85.6% accuracy for erythrocyte/leukocyte detection with the test dataset. Convolutional neural network training and comparison demonstrated that YOLOv5x for the detection of eggs and NasNetLarge for the binary image classification to detect erythrocytes/leukocytes were the best options for our digital image database.

**Conclusions:**

The development of low-cost novel diagnostic techniques based on the detection and identification of *S*. *haematobium* eggs in urine by AI tools would be a suitable alternative to conventional microscopy in non-endemic settings. This technical proof-of-principle study allows laying the basis for improving the system, and optimizing its implementation in the laboratories.

## Author summary

Urogenital schistosomiasis, categorized as a Neglected Tropical Disease (NTD) by the World Health Organization (WHO), affects approximately 150 million individuals globally, predominantly in resource-limited regions of Africa. Gold standard diagnosis relies on visually identifying of *Schistosoma haematobium* eggs in urine samples using optical microscopy. However, novel diagnostic techniques based on digital image analysis by Artificial Intelligence (AI) tools are a suitable alternative for schistosomiasis diagnosis. In this technical proof-of-principle study, a small number (n = 24) of urine sediment samples were analyzed using AI models in non-endemic settings. The study involved manually labeling *S*. *haematobium* eggs in digital images, for training YOLOv5 and YOLOv8 models for automatic egg detection, and employing MobileNetv3Large, EfficientNetv2, and NasNetLarge models for binary classification of erythrocytes/leukocytes. A robotized microscope system facilitated automated sample movement and focusing. Results indicated high precision (99.3%) and recall (99.4%) for *S*. *haematobium* detection with YOLOv5x. NasNetLarge achieved 85.6% accuracy in erythrocyte/leukocyte detection. Overall, YOLOv5x for egg detection and NasNetLarge for cell classification proved most effective. The study suggests AI-based techniques offer a cost-effective alternative to conventional microscopy for diagnosing *S*. *haematobium* infections. The automated system’s robustness and simplicity could facilitate widespread adoption in laboratories worldwide.

## 1 Introduction

**Fig 1:**
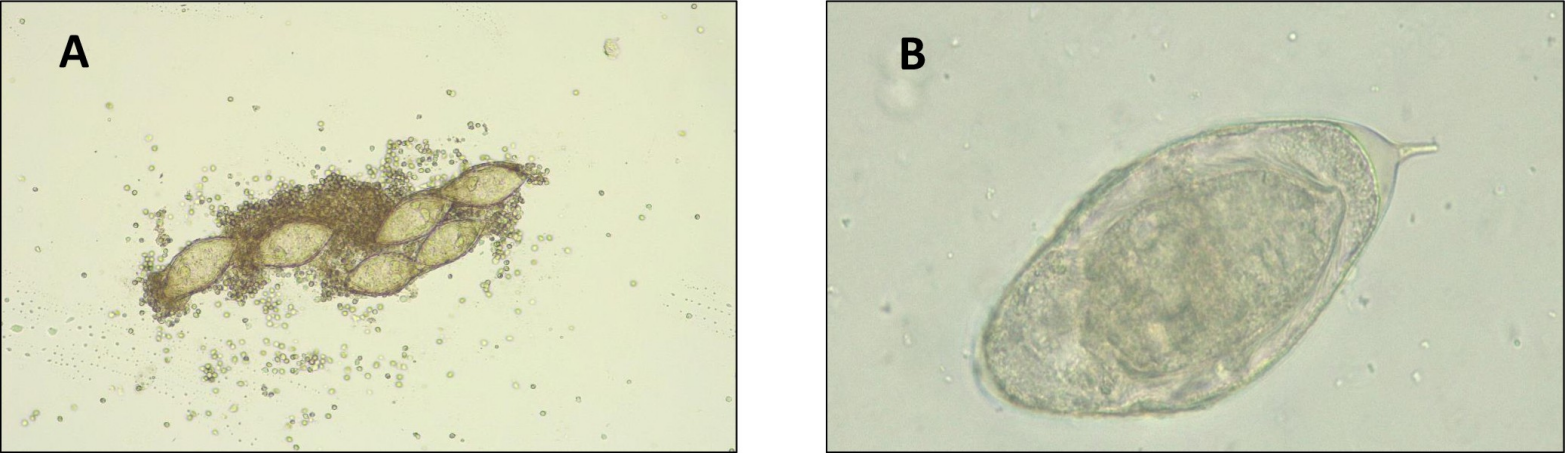
**Panel A.** *S*. *haematobium* eggs in a urine sediment sample (10x ocular; 10x objective lens). **Panel B.** *S*. *haematobium* egg in a urine sediment sample (10x ocular; 40x objective lens).

Schistosomiasis is a parasitic disease caused by *trematode* worms of the genus *Schistosoma* [1]. It affects more than 250 million people worldwide, with a high prevalence in tropical and subtropical areas [2]. Its transmission occurs by direct contact with contaminated water, in which *Schistosoma cercariae* can penetrate human skin thus initiating infection. Freshwater snails of the genera *Biomphalaria*, *Oncomelania*, and *Bulinus* act as intermediate hosts [3]. Schistosomiasis is considered a Neglected Tropical Disease (NTD) by the World Health Organization (WHO) due to its impact in resource-poor areas and its correlation with poverty [4]. The most predominant causative species are *Schistosoma haematobium* and *Schistosoma mansoni*, classified as urogenital and intestinal schistosomiasis, respectively [1]. More than 90% of schistosomiasis cases occur in the African continent, and 66% are caused by *S*. *haematobium* [5]. Some of the main countries with a high prevalence of urogenital schistosomiasis are Senegal, Nigeria, Angola, and Cameroon, among others [6–8]. Therefore, urogenital schistosomiasis has a major impact globally, with a significant incidence in the pediatric population of endemic areas [5]. Resource-poor areas near infested rivers are considered high-risk communities in which the prevalence of urogenital schistosomiasis is considerably high [9]. Digital images of *S*. *haematobium* eggs are represented in **Fig 1**. Most patients are asymptomatic, although if the disease is left untreated it can become chronic, cause hematuria and leukocyturia, and in some cases it can lead to bladder cancer [10].

According to the WHO, the gold standard diagnostic technique for schistosomiasis is the microscopic observation of parasite eggs in stool/urine samples [1]. Serological tests are also widely employed for the diagnosis of this disease [11], and molecular techniques such as real-time PCR are currently being developed to improve the sensitivity and specificity of traditional techniques [12]. However, in schistosomiasis-endemic areas, microscopy is still the most employed technique for diagnosis. Microscopic diagnosis is expert-dependent, time-consuming, and could trigger diagnostic errors due to observing large numbers of samples in short periods of time [13].

As an emulation of traditional microscopy, novel image analysis techniques based on Artificial Intelligence (AI) tools are being developed to automate the diagnostic procedure. As an example, a prototype based on AI image analysis was developed for the detection of soil-transmitted helminths and *S*. *mansoni* parasites in Kato-Katz stool thick smears [14]. Moreover, other studies demonstrate the utility of mobile phone-based microscopes for urogenital schistosomiasis diagnosis in Côte d’Ivoire [15]. Nowadays, Convolutional Neural Networks (CNNs) are the models most employed for object detection in digital image analysis [16]. Particularly, CNNs for poverty related diseases are postulated as a suitable supportive tool for the microscopic diagnosis of several diseases, such as malaria [17,18], tuberculosis [19], and NTDs [20]. Moreover, novel computational strategies, such as attention modules [21] and transformers [22], are improving the performance of traditional CNNs, opening up a new range of prospects for object detection algorithms in digital imaging. Several studies demonstrate that YOLO models have outperformed other state-of-the-art CNN models for object detection, such as Faster R-CNN and RetinaNet [23,24]. YOLOv5 and YOLOv8 used similar backbones with changes in the CSPLayer/C2f module, and they were considered the best YOLO algorithms [25]. Moreover, each YOLO model has different versions depending on its architecture, considering YOLOv-s (small) and YOLOv-x (extra-large) as suitable versions for comparison, regarding the remarkable differences in performance and speed [25]. The YOLOv8 model is newer and considered an update of YOLOv5, nevertheless, the latter is still considered the best model for object detection tasks in terms of accuracy [26].

In this study, we fine-tuned object detection algorithms (YOLOv5s, YOLOv5x, YOLOv8s, and YOLOv8x) for the automated identification of *S*. *haematobium* eggs in urine samples. A small number of biological samples from non-endemic settings were employed for digital image acquisition and labeling of parasites by a smartphone/microscope camera. CNN training and metrics comparison were performed to determine the optimal algorithm for schistosomiasis diagnosis with our image database. Binary image classification algorithms (MobileNetv3Large, EfficientNetv2, and NasNetLarge) were also trained to detect digital images with the presence of erythrocytes and/or leukocytes in urine, as a suggestive, although not exclusive, clinical sign of urogenital *S*. *haematobium* infection.

An automated robotized low-cost microscope prototype was employed for sample analysis, with X-Y movements of the slide and autofocusing. A first proof-of-principle technical study in non-endemic settings of the development of the system has been conducted, allowing to evaluate the performance and to lay the basis to enhance it in further in-field studies.

According to the state-of-the-art there are several studies describing detection methods for the automated identification of *S*. *haematobium* eggs in urine sediment samples. However, our system could contribute novelties in terms of adaptability to conventional optical microscopes and its autofocus algorithm, the usage of a smartphone device as a key controller for image acquisition and AI-analysis, and the identification of erythrocytes and leukocytes in urine for *S*. *haematobium* diagnosis orientation. Many of the published systems involve the detection of *S*. *haematobium* eggs in digital images, although the combination of all system features provide new advances in low-cost automated detection.

Thus, our image analysis strategy would join the global effort to fight against NTDs, providing clinical laboratories with novel diagnostic tools able to complement state-of-the-art traditional technologies.

## 2 Materials and methods

In this section, we describe the materials and methods used to develop the diagnostic system based on image analysis by image classification and object detection. The details of all procedures are sufficient to allow for experimentation by third parties.

### 2.1. Ethics statement

This study was conducted in accordance with the Declaration of Helsinki and approved by the Clinical Research Ethics Committee (CEIm) of the Vall d’Hebron University Hospital / Vall d’Hebron Research Institute, with reference number PR(AG)40/2023. The urine samples were not collected for the study, they were obtained from the regular clinical visits in our International Health Center and retrospectively analyzed. Formal consent was not obtained with ethics committee approval.

### 2.2. Sample preparation and observation

In this study, 24 *S*. *haematobium*-positive urine sediment samples and eight negative urine sediment samples from (i) the Drassanes-Vall d’Hebron International Health and Infectious Diseases Centre (Barcelona, Spain), and (ii) the Microbiology Department of the Vall d’Hebron University Hospital (Barcelona, Spain) were employed. Urine sediment samples were collected directly in a 100 mL recipient. Samples are left to settle for 1 hour, and 10 mL were centrifuged for 5 minutes at 15,000 rpm. Parasite eggs were searched in urine sediment samples for microbiological diagnosis. Standard objective lenses, 10x and 40x, were employed to perform a large-scale observation and confirm the diagnosis, respectively. Between 1–20 images of different microscopy fields were acquired from each sample, considering parasite densities, presence/absence of erythrocytes and leukocytes in urine, and number of *S*. *haematobium* eggs per sample. This range of image numbers avoids an imbalance in the training of the neural networks by employing a stipulated number of data. Biological samples were obtained from symptomatic/asymptomatic patients from *S*. *haematobium*-endemic areas, mainly migrants, and Visit Friends and Relatives (VFR), following our reference center’s protocols [27]. Most individuals came from Sub-Saharan Africa region, mainly from Gambia, Mali and Senegal. Regarding epidemiological studies, the prevalence of *S*.*haematobium* infection in these countries is between 9% to 10.2% [28–32]. Microscopic examination of clinical samples was performed following WHO sample observation statements for *S*. *haematobium* diagnosis [32]. Samples were observed at most 48h after extraction and discarded, following our International Health Laboratory protocols.

### 2.3. Image acquisition

**Fig 2:**
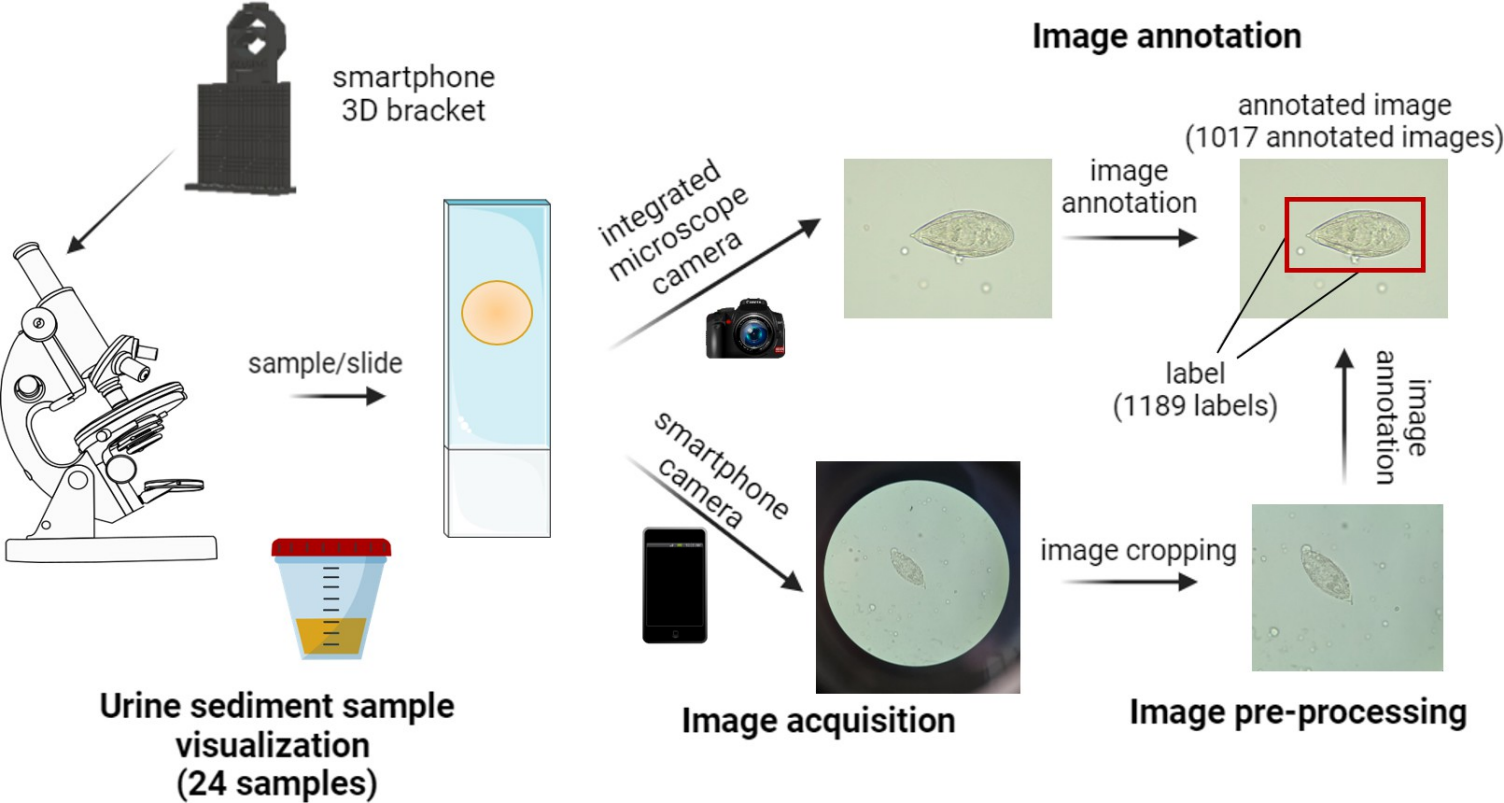
Representative scheme of sample visualization, image acquisition, image pre-processing, and image annotation procedures. Sample/slide, image, annotated image, and label are represented. *Schistosoma haematobium* digital images were acquired with an integrated camera/smartphone camera in the Microbiology Laboratory of the Vall d’Hebron Drassanes International Health and Infectious Diseases Centre. Illustrations were obtained from open source resources.

Digital images of each microscopic Field of View (FoV) were acquired with an integrated Leica ICC50W camera attached to a Leica DM750 microscope (5.0MP / 2592 x 1944 pixels) and consecutively with the camera of a Samsung Galaxy S20 smartphone device (64MP, 0.8μm, f/2.0, OIS / 3024 x 4032 pixels). Smartphone-acquired images were captured using an ocular adapter for smartphone attachment to the microscope. A 3D adapter bracket attached to the ocular lens of the microscope was used to standardize the image-capturing procedure with the smartphone device. Both, the integrated camera and smartphone-acquired images were captured by the visualization of urine sediment samples with a Leica DM750 microscope lens with 100x and 400x total magnification (10x ocular; 10x and 40x objective lens). Image acquisition is represented in **Fig 2**.

### 2.4. Image pre-processing

Images acquired with a smartphone device were cropped to highlight the central area of interest of the image and to eliminate black borders due to ocular lens attachment. Cropping was performed to remove the outer edges without losing any information (**Fig 2**). Original smartphone images were cropped automatically (Python script) to obtain a 4:3 image in the center and subsequently rotated 90° for horizontal image reorientation. With this procedure, it is possible to crop an image regardless of its dimensions and the number of pixels as proportions were used to perform the cropping. The cropped images have the same 4:3 image proportion as those acquired with the microscope-integrated camera. Cropping confers a re-composition of the image that may positively affect the final results, providing a clearer image and removing elements irrelevant to the prediction and identification functions of the neural networks, as mentioned in the previous literature [33].

### 2.5. Image annotation and classification

Digital images were labeled by laboratory professionals from the Drassanes-Vall d’Hebron International Health and Infectious Diseases Centre. For image labeling, the area of interest was selected by creating a bounding box with the object inside (**Fig 2**). This bounding box defines the label. *S*. *haematobium* eggs (viable and calcified) were labeled in digital images using Annotation App software. Labeling eggs with bounding boxes was required to train the object detection algorithms based on CNNs. Once labeling was finished, the Annotation App software created a *json* type file with labels linked to the original image file, in which the coordinates of the labeled objects are specified (annotated images). Whole-image classification was performed by creating two subgroups of images depending on whether erythrocytes/leukocytes were present or not in urine sediment samples.

### 2.6. Binary classification algorithms for hematuria and leukocyturia detection

Whole-image binary classification models (MobileNetv3Large [34], EfficientNetv2 [35], and NasNetLarge [36]) were employed to automatically classify digital images in two subgroups. Digital images with or without the presence of erythrocytes/leukocytes in urine samples, with 100x magnification (10x ocular; 10x objective) were manually classified by three clinical laboratory experts from the Vall d’Hebron International Health and Infectious Diseases Centre. The image database was divided, allocating 90% for training/validation and 10% for testing. Images were resized by default to 331x331 pixels for NasNetLarge, 224x224 pixels for MobileNetv3Large, and 300x300 pixels for EfficientNetv2, and trained for 30 epochs and batch size 20. Only 100x magnification images (10x ocular; 10x objective) were employed for binary classification algorithm training.

### 2.7. Object detection algorithm training and comparison analysis

CNN object detection models were fine-tuned with our *S*. *haematobium*-labeled digital image database. You Only Look Once (YOLO) [37] versions 5s, 5x, 8s, and 8x were trained. YOLO was considered an optimal algorithm for object detection tasks, with several studies in the area of diagnosis [25,38,39]. The YOLO versions were also crucial, considering YOLO “s” (small) as a fast and efficient alternative and YOLO “x” (extra-large) as a more accurate algorithm [25,39]. The *Schistosoma* image database was divided, allocating 80% for training, 15% for validation, and 5% for testing. Images were resized by default to 640x640 pixels and CNNs were trained for 30 epochs and batch size 16. Images were organized randomly considering the proportions, and test subset images were unseen by the CNN model to avoid unreliable results and preserve patient-level structure [40]. Neural networks were pre-trained with the Common Objects in Context (COCO) dataset [41]. Object detection CNNs were compared using the analytical metrics of precision, recall, F-score, and mAP0.5. Two consecutive image datasets were employed for CNN training and evaluation. An initial image dataset containing 491 images was employed, and thereafter, a second dataset containing 1017 images, which also included the images of the initial database (491). The number of images employed for CNN training was determined considering supervised single-class classifiers performance and other similar studies, although the minimum amount of labels to obtain reliable results depends on the labels quality and classifier architecture [42,43].

### 2.8. Statistical analysis

Statistical analyses were performed to determine significant differences between validation and test subset performance for each CNN model. Metric means were calculated individually for each CNN model. To evaluate significant statistical differences between CNN models, a paired t-test analysis (*p*-value<0.05, t-value > -2 or 2), mean (M), and standard deviation (SD) were employed. The same statistical analysis was employed for binary classification algorithms. The IBM SPSS software statistics environment was used.

### 2.9. Microscopy automation system

The system [44] was employed to automate a conventional Leica DM750 optical microscope for malaria diagnosis. This technology can autofocus the image/FoV and guide the automated movements of the slide through the X-Y axis of the microscope. All the diagnostic technology is embedded into a smartphone/computer application, responsible for acquiring the images, automating microscope auto-focus and stage movements, and using CNN algorithms for parasite detection. The system is designed with 3D-printing technology and does not need an internet connection or an electrical power supply. Smartphone-based, adaptability and auto-focus results were previously published [44].

## 3 Results

### 3.1. *Schistosoma haematobium* urine sediment image database analysis

*S*. *haematobium* urine sediment samples, digital raw images, annotated images, and labels were analyzed. A total of 1017 annotated digital images were imported into the database for further CNN training and algorithm generation. A total of 1189 labels identifying *S*. *haematobium* eggs (calcified/non-calcified) were annotated in digital images. Of the 1017 digital images, 744 were acquired with the LEICA ICC50W integrated microscope camera, and 273 were acquired with the Samsung Galaxy S20 smartphone camera. For the detection of erythrocytes and/or leukocytes in urine, 762 images were acquired with the integrated microscope camera LEICA ICC50W and manually classified as urine samples with (493 images) or without (269 images) erythrocytes/leukocytes, respectively. A summary of the urine sediment sample image database information is shown in **Table 1**.

**Table 1:** Summary of the urine sediment sample image database.

Category		Sub-total	Total
**Sample source**	Drassanes-Vall d’Hebron International Health and Infectious Diseases Centre (Barcelona, Spain)	24 (samples/patients)	24 (samples/patients)
**Image acquisition type**	Microscope integrated camera (ICC50W Leica)	744 (annotated images)	1017 (annotated images)
	Smartphone camera (Samsung Galaxy S20)	273 (annotated images)	
**Image magnification**	10x ocular lens and 10x objective lens	500 (annotated images)	1017 (annotated images)
	10x ocular lens and 40x objective lens	517 (annotated images)	
**Annotation category**	*Schistosoma haematobium* eggs	1165 (labels)	1189 (labels)
	*Schistosoma haematobium* calcified eggs	24 (labels)	
**Binary image classification**	Presence of erythrocytes and/or leukocytes in urine	493 images	762 images
	Non-presence of erythrocytes and/or leukocytes in urine	269 images	
**Negative sample validation**	Non-presence of *Schistosoma haematobium* eggs in urine	8 (samples/patients)	400 images

### 3.2. Convolutional Neural Network performance comparison for *S*. *haematobium* egg detection

CNN models were trained with our *S*. *haematobium* image database and compared to evaluate their performance in a test subset. **Table 2** shows the most relevant metrics of the YOLOv5s, YOLOv5x, YOLOv5x-DA, YOLOv8s, and YOLOv8x CNNs trained with 491- and 1017-image databases. Overall analysis confirms that the 1017-image database provides higher metric results for CNN training than the 491-image database, as expected. Considering the 1017-image database training, precision analysis shows optimal values with the YOLOv5 model, with 99.3% for YOLOv5x and 97.1% for YOLOv5s. Recall analysis demonstrates a 99.4% rate for YOLOv5x and 97.2% for YOLOv5s. Consequently, F-score analysis demonstrated optimal values with the YOLOv5x (99.3%) and YOLOv5s (97.1%) models. Mean average precision (mAP0.5) analysis shows higher values for YOLOv5x, YOLOv5s, and YOLOv8s with 99.4%, 98.8%, and 98.7% respectively, all with the 1017-image database. Overall metric analyses in terms of precision, recall, F-score, and mAP0.5 indicated that the best CNN model for *S*. *haematobium* detection in urine sediment samples with our image database was YOLOv5x (1017).

**Table 2:** Summary of Convolutional Neural Network training and performance parameters with the test image dataset. DA: Data augmentation, mAP: mean average precision, YOLO: you only look once.

Neural Network model	Epochs	Precision (%)	Recall (%)	F-score (%)	mAP0.5 (%)	Images
YOLOv5x	30	92.3	73.3	81.7	81.7	491
YOLOv5x - DA	30	88.2	72.4	79.5	85.3	491
YOLOv8s	30	94.3	97.0	95.6	97.3	491
YOLOv8x	30	95.3	89.8	92.5	96.8	491
YOLOv5s	30	97.1	97.2	97.1	98.8	1017
YOLOv5x	30	**99.3**	**99.4**	**99.3**	**99.4**	1017
YOLOv8s	30	96.3	96.5	96.4	98.7	1017
YOLOv8x	30	96.3	95.1	95.7	96.6	1017

**Fig 3:**
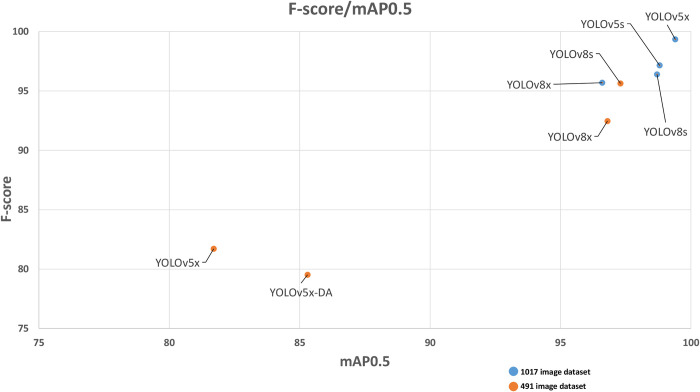
Graphical representation of F-score/mAP0.5 of the different CNNs trained on the test dataset. Orange dots represent the performance of CNNs trained with the 491-image dataset. Blue dots represent the performance of CNNs trained with the 1017-image dataset.

**Fig 4:**
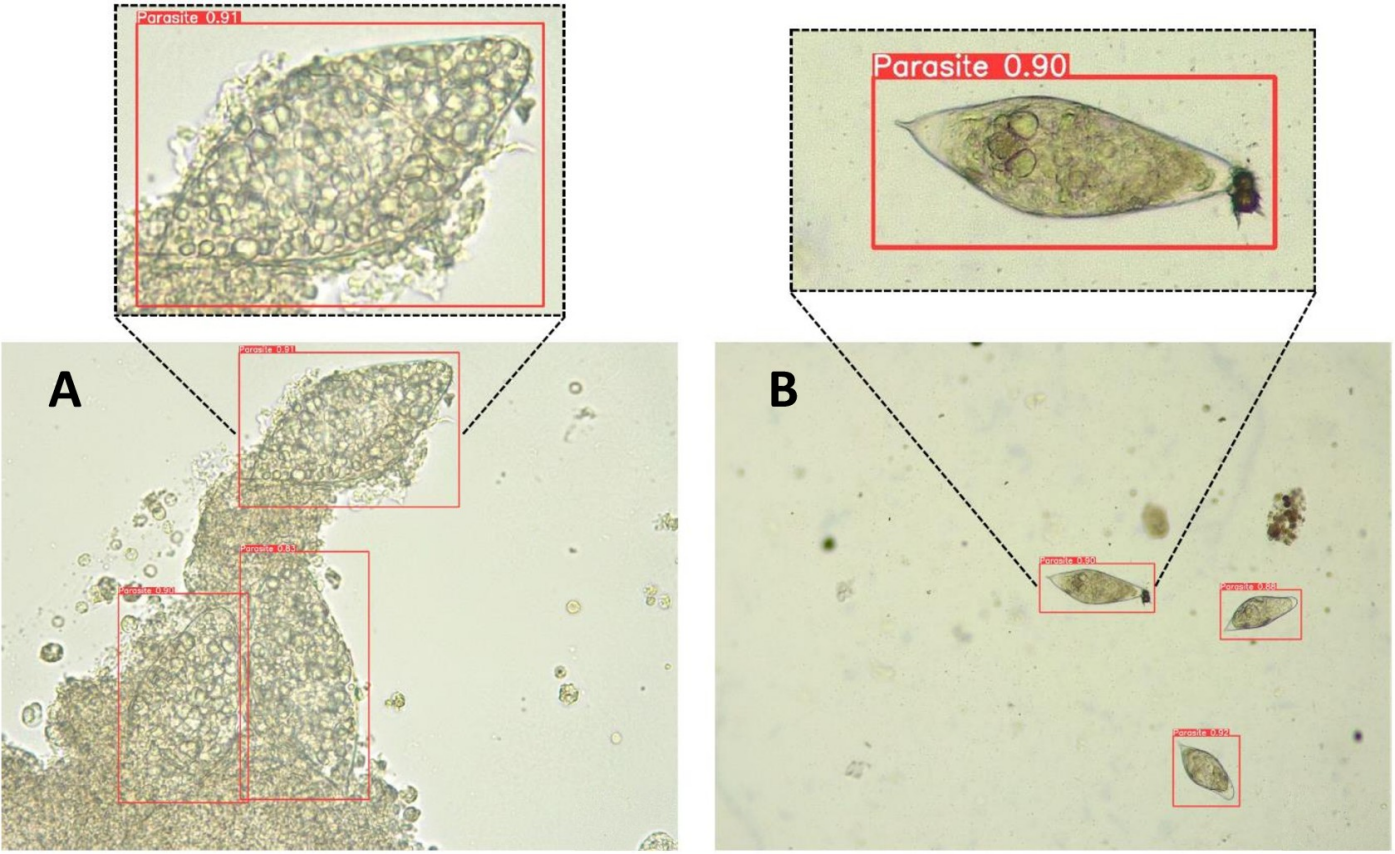
**Panel A**. Digital image (400x) of a urine sediment sample with hematuria and leukocyturia and three *Schistosoma haematobium* eggs detected with the YOLOv5x trained model. **Panel B**. Digital image (100x) of a urine sediment sample with three *S*. *haematobium* eggs detected with the YOLOv5x trained model.

To determine differences between neural network performances for *S*.*haematobium* egg detection in digital images, a statistical analysis was conducted considering the test data subset. As expected, models trained with a larger image dataset (1017) show considerably higher performance when compared with the previous smaller image dataset (491) (*p*<0.05). The impact of the size of the database was studied, even though it might seem obvious, to analyze whether results using more images were necessary. Results of the paired t-test indicated that there were significant statistical differences between YOLOv5x–DA-491 (M = 42.3, SD = 41.7) and YOLOv5x-1017 (M = 51.3, SD = 51), t = 2.4, *p*<0.05. Moreover, results of the paired t-test indicated that there were significant statistical differences between the YOLOv5-491 (M = 42.8, SD = 40.1) and YOLOv5-1017 trained versions (M = 50.7, SD = 49), t = 3, *p*<0.05). These data demonstrate the performance gain due to the higher number of data/images for training the YOLOv5x model. In contrast, there are non-statistically significant differences between models YOLOv8s-491 and YOLOv8s-1017 (*p*>0.05) and models YOLOv8x-491 and YOLOv8x-1017 (*p*>0.05). The performance gain due to the image dataset increase was not obtained with model YOLOv8s or YOLOv8x. The optimal results of YOLOv8 with a relatively small image database (491) demonstrate the efficiency of the neural network compared with YOLOv5. F-score values, which are the harmonic mean between precision and recall, and mAP0.5, provide valuable information to determine the best model for our image dataset. **Fig 3** shows the correlation between the F-score and mAP0.5 values of the multiple trained neural networks. The dots represented in the top right part of the graph reflect optimal performance. Overall analysis and comparisons demonstrate that the YOLOv5x model is the most optimal CNN for our digital image dataset, although the YOLOv8 models are more efficient in terms of training and image database size. Differences between small “*s*” and large “*x*” CNN architectures were not statistically significant (*p*>0.05). Image identification was represented in **Fig 4**.

### 3.3. Binary image classification performance for erythrocyte/leukocyte detection in urine sediment images

Whole-image classification algorithms were trained with our *S*. *haematobium* image database and compared to evaluate their performance. **Table 3** shows the most relevant metrics to evaluate the performance of the MobileNetv3Large, EfficientNetv2, and NasNetLarge models. There were no statistically significant differences between the performance of these three models (*p*>0.05), or between validation (M = 85.5, SD = 3.9) and test (M = 82.4, SD = 4) subsets in terms of accuracy results (*t* = 1.8, *p* > 0.05). However, NasNetLarge was considered the best option for the erythrocytes/leukocytes image classification task with an 85.6.0% accuracy, followed by the MobileNetv3Large and EfficientNetv2 models, with 83.7% and 77.9% accuracy, respectively.

**Table 3:** Summary of binary image classifier training and performance parameters on validation and test image datasets.

Image classifier model	Epochs	Batch size	Validation Accuracy	Test Accuracy
MobileNetv3Large	30	20	83.3	83.7
EfficientNetv2	30	20	83.3	77.9
NasNetLarge	30	20	90.0	85.6

### 3.4. Negative sample validation

Analysis and validation tests were performed to evaluate the reliability of the YOLOv5x-1017 model trained with confirmed negative samples. Urine sediment samples (n = 8) with a negative microscopic examination result for *S*. *haematobium* eggs were employed. Samples were observed and validated by professional microscopists with proven experience in parasitological diagnosis. Five microscope slides of each sample were prepared to observe the whole urine sediment. A total of 400 images were acquired from urine sediment samples and analyzed by the trained YOLOv5x model (confidence threshold value = 0.7). These negative sample images were not used for CNN algorithm training. After analysis, 394/400 (98.5%) were negative (true-negative) for *S*.*haematobium* infection, and six false-positive results were reported (epithelial cells, urine crystals, and artifacts). All false-positive images were from a single urine sediment sample.

### 3.5. Testing analysis with other image databases

To determine the robustness of our trained models, we tested the considered optimal model, YOLOv5x, with digital images from other sources. However, there are no publicly available large state-of-the-art *S*. *haematobium* urine sediment databases to compare our results. As an alternative, publicly available single images were obtained from contrasted cited sources [23–26,45–47]. We selected and employed images with different input sizes (from 1600 x 1200 pixels to 400 x 300) and different image weights (from 895 KB to 25.9 KB), ensuring a representative sample (n = 19). Digital images with parasite eggs were empirically tested with our fine-tuned YOLOv5x model with positive results (27/27 eggs detected, average detection probability value = 0.79).

### 3.6. Automated microscope for diagnosis and smartphone software application

To automatically perform an autonomous detection of *S*. *haematobium* eggs in urine sediment samples, we designed a fully automated low-cost robotized microscope. Three-D polylactic acid pieces for microscope automation were built with an Ender 3-Pro printer. Automation allows the emulation of optical microscope movements via 9G servo motors and an Arduino MKR Wi-Fi 1010 controller. X-Y and Z (auto-focus) through the microscope slide permit the system to acquire images of different FoVs for further CNN detection. To auto-focus each FoV, a Variance of the Laplacian algorithm was employed [48]. Considering Schistosomiasis-endemic areas, which are usually resource-poor settings, the microscope was designed with low-cost materials and does not require continuous electric power supply. However, its implementation in resource-poor settings should be tested. Portable solar batteries grant the system energy autonomy if this is not available in the laboratory. In addition, the 3D pieces were designed with a range of measures as universal adapters for the vast majority of optical microscopes. A smartphone device controls the system’s movements, via servo motors, through the X-Y-Z axis of the microscope. The Arduino controller is connected to the smartphone device via a Bluetooth low-energy connection, which additionally acquires the images and auto-focuses the sample for further CNN object detection analysis. The whole diagnostic process is integrated into a smartphone-based application developed with Android Studio Programming environment v.2021 [49]. This automated system has already been employed by our research group for the automated detection of *Plasmodium* trophozoites and leukocytes in thick blood smear samples for malaria diagnosis [50].

## 4. Discussion

Schistosomiasis diagnosis by microscopic examination of urine and stool samples is still the gold standard technique and is widely used in resource-poor areas. However, the continuous reduction in microscopy parasitologist experts [13] requires the development and implementation of novel diagnostic techniques for the diagnosis of schistosomiasis and other NTDs. In this study, we have developed a novel diagnostic technique based on the automatic detection of *S*. *haematobium* eggs in urine sediment samples using AI tools and a robotized low-cost 3D microscope system. Moreover, a first proof-of-principle study was performed to evaluate its detection potential and implementation in non-endemic settings.

Following WHO guidelines for Schistosomiasis diagnosis, we have employed urine sediment samples and consequent image acquisition [32]. Clinical urine sediment samples are crucial to properly acquire digital images of *S*. *haematobium* eggs, train CNN models, and finally emulate a traditional microscopic diagnosis by AI image analysis techniques. *In vivo* and *in vitro* cultures of *S*. *haematobium* for laboratory-kept parasite lifecycle were described in the mid-1960s [51]; according to our knowledge, there are no publications describing their use for digital image acquisition and database generation for further CNN training. The conservation of fresh *S*. *haematobium* eggs can be difficult due to degradation. It would be optimal to capture images of *S*.*haematobium* eggs *in situ* at field laboratories, in order to obtain a more robust and representative database. As a limitation of the study, (i) images should be acquired at most 24/48 hours after sample collection, if not kept at 4°C [52]. Moreover, (ii) system development was performed in non-endemic settings, resulting in sample collection difficulties due to the lower number of cases received in comparison with endemic regions. Sample size was small, therefore further investigation is needed to obtain more robust and conclusive results. Finally, (iii) a system validation should be pursued in *S*. *haematobium* endemic regions, to evaluate its performance in such environments. Although the system has been designed for its implementation in resource-poor settings, it has initially been evaluated in non-endemic areas. Therefore, it is crucial to conduct pilot tests in the field and further studies to assess diagnostic performance in such environments. Some of the system aspects that would allow its implementation in rural areas with few resources are: its adaptability to conventional optical microscopes, its portability, the low-cost of the system and the non-internet connection required.

As an attribute of the study, the detection of *S*. *haematobium* basal stage and calcified eggs was implemented to detect both egg forms. Calcified eggs are typically found in chronic bladder infections and, sometimes, basal-stage eggs were not present, making their detection essential for a proper diagnosis [53]. One of the main strengths of our study is the training and comparison of different YOLO neural network models. The continuous advancements in CNN development and improvement are generating more efficient models for object detection. The YOLOv5 and YOLOv8 models were considered optimal options in terms of accuracy and inference speed for object detection tasks [38]. Our results have shown that YOLOv5x was the best option for *S*. *haematobium* egg detection with our digital image database (**Table 2**). The improved YOLOv8 model shows a more efficient performance in terms of training in comparison with YOLOv5; YOLOv8 models trained with the 491-image dataset demonstrated optimal performances for all descriptive parameters. However, when the 1017-image dataset was employed, the YOLOv5 models demonstrated better performance compared with YOLOv8 (**Fig 3**). Other studies have compared the performance of the YOLOv5 and YOLOv8 models with very variable results [39,54]. Sary et al. 2023, compared the performance of both models for human detection in aerial images, showing better precision and F-score values for YOLOv8 and higher recall values for YOLOv5 [55]. Nevertheless, Sirisha et al. 2023, reported that the YOLOv5 model has a higher mAP0.5 value compared with other YOLO versions [38]. In addition, both YOLOv5 and YOLOv8 were employed for diagnostic tasks such as detecting developmental dysplasia of the hip in radiography images [56], and localization of dermoscopic structures [57], conferring the algorithms a contrasted efficacy for image-based diagnosis.

Another important aspect of this study was the identification of erythrocytes and leukocytes in the urine with the NasNetLarge model, allowing a diagnostic orientation due to the high correlation between the presence of erythrocytes in urine and *S*. *haematobium* eggs. It is important to note that haematuria may appear in multiple clinical situations; however, in schistosomiasis diagnostic protocols, a high correlation rate (78%) was observed, as shown in other studies [58]. We could not confirm that haematuria and leukocytes are specific to the infected individual, although if they were detected through images we could continue observing several sample replicas to find parasite eggs. Moreover, the NasNetLarge model had provided contrasted results for binary image classification with other similar diagnostic tasks, such as melanoma skin lesion detection and classifying diabetic retinopathy severity in digital images [59,60].

Other studies demonstrated the applicability of object detection algorithms for *Schistosoma* egg detection in stool and urine samples [61–64]. Before the irruption of CNNs, other strategies, such as the multi-class support vector machine (MCSVM) for parasite egg classification, were developed with an overall performance of 97.7% [62]. However, CNNs improved traditional image object detection in terms of computational potential, speed, and performance. Werd et al. 2022, developed an affordable AI-based system for the detection of soil-transmitted helminths and *S*. *mansoni* eggs in stool samples with the R-FCN ResNet101 COCO model [14]. They obtained an F-score of 88.9% for *Schistosoma* detection in stool samples, in comparison with the 99.3% value in urine samples of our fine-tuned YOLOv5x model. However, we must consider that the number of artifacts in stool samples is much higher than in urine samples. In addition, the number of images employed for CNN training was larger in our study. The difficulty in obtaining parasite egg digital images has led to several studies using data augmentation (DA) strategies [63]. Oliveira et al. 2022, used a DA strategy (66 original images) for *S*. *mansoni* egg detection in microscopy images with the Faster R-CNN model, showing a 76.5% precision [64]. Nevertheless, with our image dataset, we did not observe any statistically significant differences between DA and non-DA training, as shown in **Table 2**. As a breakthrough, Oyibo et al. 2022, developed an optical automated system based on AI for the detection of *S*. *haematobium* and *S*. *mansoni* eggs in urine and stool, respectively, for implementation in resource-poor settings [65]. Moreover, they mainly used non-clinical samples for UNET model training, which may interfere with its final performance. In addition, Schistoscope 5.0 shows an 80.1% sensitivity and 95.3% specificity for *S*. *haematobium* detection. However, it is a non-optimal system for transportation and needs to be suitable for the WHO target product profile for new diagnostics [66]. Its contribution is a milestone for automated schistosomiasis diagnosis and could be an alternative to conventional diagnosis in coalescence with other similar studies. Additionally, they have demonstrated that the employment of negative urines from endemic regions is crucial in validation studies, due to the presence of abundant artifacts that could be identified as parasite eggs [66]. Therefore, in our study, a negative sample validation should be pursued with a large amount of samples (>8) to evaluate the system performance in the field. Oyibo et al. 2023 also developed a detection framework for the diagnosis of urogenital schistosomiasis in microscopy images with from low-resource settings. The framework demonstrates a clinical sensitivity, specificity, and precision of 93.8%, 93.9%, and 93.8%, respectively, using results from an experienced microscopist as reference [67]. As another example, Coulibaly et al. 2023 performed a community based schistosomiasis screening program with a smartphone-based AI device showing 85.7% sensitivity and 93.3% specificity [15]. Its design is also 3D-printed, innovative and portable; although there are some differences with our system in terms of adaptability to other smartphone devices or microscopes. An alternative to using CNN-based models such as YOLO would be to use Vision Transformer models. However, they require larger model sizes, greater memory, and larger databases [68]. Since our database is relatively small, we decided to use CNN-based models, which provide excellent results. AiDx multi-diagnostic microscope was tested in 17 communities in Abuja, Nigeria; showing 89% sensitivity and 99% specificity for the identification of *S*. *haematobium* eggs in urine with the fully automated AiDx Assist mode [69].

Our system has proven to be robust in our microbiology laboratory (non-endemic settings) and should be validated for its implementation in resource-poor settings for further evaluation. However, developmental stages, such as the ones presented in this manuscript, were crucial to evaluate the reliability of the system before a diagnostic validation, and were the basis for further in-field studies. Overall, we believe that this novel automated low-cost AI-based diagnostic system for parasite detection could join the global effort to fight NTDs and poverty-related diseases worldwide.

## 5. Conclusions

Automated schistosomiasis detection is a big challenge to support and optimize traditional microscopic diagnosis. Deep learning-based diagnostic techniques would help improve diagnostics and could be a suitable tool for the training and education of professionals. NTDs, such as schistosomiasis, are being significantly overlooked by national and international health organizations; therefore, novel solutions to improve the management of these diseases would be of significant benefit to the most vulnerable affected populations. Comparison of different YOLO object detection models allowed us to choose the best algorithm for our detection, ensuring that these results could be replicated in other similar studies. The automation of the entire process by robotization with 3D parts and servomotors for a conventional optical microscope allows the emulation of the X-Y slide movements and sample auto-focus. The integration of image analysis and microscope automation provides the system with attributes that render it accessible, affordable, and highly autonomous. Moreover, the low-cost and easy-to-handle technology was designed to be implemented in any laboratory, regardless of their resources. To this end, it is important to understand the needs related to the diagnosis of schistosomiasis and other NTDs in the field and to jointly develop solutions for the correct implementation of new AI-based technologies. In conclusion, we are getting closer to developing an automated diagnostic system for schistosomiasis diagnosis based on AI tools, to fight NTDs and other poverty-related diseases.

## Supporting information

S1 Dataset Training dataset configuration. (YAML)

S1 Table Fig 3 values. (XLSX)

S2 Table Negative sample validation values. (XLSX)
