# Correction: Development of an automated artificial intelligence-based system for urogenital schistosomiasis diagnosis using digital image analysis techniques and a robotized microscope

**DOI:** 10.1371/journal.pntd.0013643

**Published:** 2025-10-28

**Authors:** Carles Rubio Maturana, Allisson Dantas de Oliveira, Francesc Zarzuela, Edurne Ruiz, Elena Sulleiro, Alejandro Mediavilla, Patricia Martínez-Vallejo, Sergi Nadal, Tomàs Pumarola, Daniel López-Codina, Alberto Abelló, Elisa Sayrol, Joan Joseph-Munné

There are errors in the Funding statement. The correct Funding statement is as follows: This research was funded by the Microbiology Department of Vall d’Hebron University Hospital, the Computational Biology and Complex Systems Group, Physics Department of the Universitat Politècnica de Catalunya, the Cooperation Centre of the Universitat Politècnica de Catalunya (CCD-UPC) and the Signal & Data Processing Research Group at TecnoCampus. In addition, this study has been funded by Instituto de Salud Carlos III trough the project “PI20/00217” to JJ-M (Co-funded by European Regional Development Fund: “A way to make Europe”), Grant 2021-SGR-00582 funded by Agència de Gestió d’Ajuts Universitaris i de Recerca (Generalitat de Catalunya), Spanish national plan PEICTI, project WaterWritten (PID2023-14664OBI00 to ESay) and Ministerio de Ciencia e Innovación (PID-2022-139216NB-I00,MCIN/AEI/10.13039/501100011033 to DL-C). All funders had roles in study design, data collection, statistical analysis and manuscript preparation and edition.
